# Plasmonic enhancement of aqueous processed organic photovoltaics†

**DOI:** 10.1039/d1ra02328d

**Published:** 2021-05-25

**Authors:** R. Chowdhury, L. Tegg, V. J. Keast, N. P. Holmes, N. A. Cooling, B. Vaughan, N. C. Nicolaidis, W. J. Belcher, P. C. Dastoor, X. Zhou

**Affiliations:** Department of Physics, School of Mathematical and Physical Sciences, The University of Newcastle Callaghan NSW 2308 Australia Xiaojing.zhou@newcastle.edu.au; Centre for Organic Electronics, Faculty of Science, The University of Newcastle Callaghan NSW 2308 Australia; Australian Centre for Microscopy and Microanalysis, University of Sydney NSW 2006 Australia

## Abstract

Sodium tungsten bronze (Na_*x*_WO_3_) is a promising alternative plasmonic material to nanoparticulate gold due to its strong plasmonic resonances in both the visible and near-infrared (NIR) regions. Additional benefits include its simple production either as a bulk or a nanoparticle material at a relatively low cost. In this work, plasmonic Na_*x*_WO_3_ nanoparticles were introduced and mixed into the nanoparticulate zinc oxide electron transport layer of a water processed poly(3-hexylthiophene):phenyl-C_61_-butyric acid methyl ester (P3HT:PC_61_BM) nanoparticle (NP) based organic photovoltaic device (NP-OPV). The power conversion efficiency of NP-OPV devices with Na_*x*_WO_3_ NPs added was found to improve by around 35% compared to the control devices, attributed to improved light absorption, resulting in an enhanced short circuit current and fill factor.

## Abstract 2

Plasmonic Na_*x*_WO_3_ nanoparticles were introduced to aqueous processed organic photovoltaics with 35% device enhancement.

## Graphical abstract

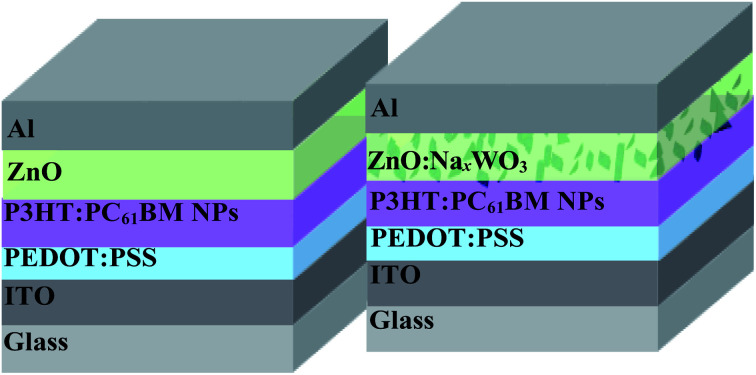

## 1 Introduction

To meet the environmental, socio-economic and ethical sustainability demands of a future energy system necessarily requires that this energy system be renewable and sustainable.^1,2^ Within this framework, solar energy (compared to other sources of renewable energy) offers a cost effective, non-polluting, sustainable, silent solution that has the capacity to supply efficient power at large scale.^3^ Over the past few decades, organic photovoltaics (OPVs), based on the bulk-heterojunction (BHJ) structure, have been developed into a promising technology due to their intrinsically low manufacturing cost and eco-friendly fabrication process through roll-to-roll printing techniques.^4,5^ Additionally, several life cycle cost analysis reports have confirmed the ability of OPV technology to contribute cost-effectively to the global energy supply.^6,7^

The main drawback of current BHJ-OPV technology is the synthesis of the photo-active layer from hazardous chlorinated and/or aromatic solvents such as chloroform (CF), chlorobenzene (CB) and dichlorobenzene (DCB). In response to these concerns, researchers have been working on non-toxic or ‘green’ solvent ink formations for OPV application.^8–11^ Non-toxic solvents, in particular, aqueous nanoparticulate organic photovoltaics (NP-OPV) have been investigated in our group since last ten years and the device efficiency has steadily increased from 1.4% to 3.3%.^12–15^ Other research groups using different donor–acceptor materials have reported NP-OPV device performance in the range of 1.9% to 3.8%.^16,17^ More recently, the efficiency of aqueous NP-OPV devices has reached 7.5% through the use of removable surfactants.^18^

Nevertheless, aqueous processed OPVs are still much less efficient than those of conventional BHJ OPVs. Previous work to improve device efficiency has focussed on investigating the donor–acceptor mixed NP domain morphology and its associated photo-physics.^19^ This research identified the origin of the poor performance of these NP-OPV devices to be: (1) the presence of excess surfactant in the photoactive layer; (2) poor wetting of aqueous nanoparticle inks resulting in low film quality in the photoactive layer; (3) improper distribution of the donor and acceptor material domains in the photoactive layer, leading to poor charge generation.^18,20^

A number of studies have already been undertaken to address these issues. For instance, removing the excess of surfactant from the NP ink and forming better quality of films by optimising the dialysis strategy and utilising the thermal treatment has been reported.^18,21^ In addition, a new rapid evaporation technique was introduced by Marks *et al.* to form highly intermixed donor–acceptor film morphology.^22^ However, the issue of poor charge generation in NP-OPV devices has yet to be addressed and requires, through careful nanoscale engineering, the development of new NP-OPV structures that are able to enhance charge generation and extraction.

Due to the low carrier mobility of conducting polymers^23^ and short exciton lifetime,^24^ the optimal thickness of the active layer of BHJ-OPVs is limited to 100 nm or less.^25–27^ Hence, this thin active layer limits the light absorption and consequently affects the photo-conversion efficiency. The introduction of plasmonic NPs into BHJ-OPVs is commonly used to improve their light absorption and charge generation by enhancing the light scattering either within the active layer or at the active layer/electrode interface, which in turn increases the length of the optical path thus improving light absorption in the photoactive layer.^28–32^ Moreover, plasmonic nanoparticles can be made into a suspension that is compatible with the solution processing techniques used for BHJ-OPV fabrication.^33–35^ To date, a number of different plasmonic nanoparticles, such as Au, Ag, graphene oxide with Au spheres, *etc.*, have been reported as plasmonic nanoparticles for BHJ-OPVs.^36–38^

Recent work has assessed the feasibility of Na_*x*_WO_3_ as an alternative plasmonic material since it exhibits strong plasmonic resonances compared to Au and offers simple nanofabrication at low cost.^39,40^ In this work, plasmonic structured NP-OPV devices have been fabricated to study the light–matter interaction in the nanoscale region between metal oxide plasmonic nanoparticle and water processed photo-active layer. A combination of microscopic and film physical/chemical spectroscopic techniques have been used to elucidate the multilayer film morphology of Na_*x*_WO_3_ NP-OPV devices and the effect of plasmonic nanoparticles on their performance.

## 2 Experimental details

### 2.1 Synthesis of Na_*x*_WO_3_ nanoparticles

Na_*x*_WO_3_ nanoparticles were prepared using a modification of furnace-assisted method of Straumanis,^41^ as described previously.^42^ Briefly, powders of sodium tungstate dihydrate (Na_2_WO_4_·2H_2_O, VWR, >99%), tungsten(vi) oxide (WO_3_, Sigma-Aldrich, 99.9%) and tungsten (Aldrich, ≥99.9%) were mixed proportionally to target *x* = 0.81. The powders were crushed and mixed using mortar and pestle, pressed under ≈20 MPa into a pellet, then heated in a vertical tube furnace at 875 °C for 3 min under flowing N_2_. The sample was then milled in a high-energy ball mill for 1 h, with a 1 : 5 charge ratio of 2–5 mm steel balls. X-ray diffraction (XRD) patterns of the Na_*x*_WO_3_ nanoparticles were collected using a Philips X'Pert MPD XRD fitted with a Cu Kα anode. Patterns were collected across 5° ≤ 2*θ* ≤ 90° in 0.013° steps, with a total acquisition time of ≈60 min. From the Rietveld analysis of the patterns, the sample purity was determined using the method of Hill and Howard,^43^ and the Na content of the Na_*x*_WO_3_ phase was found using the relationship of Brown and Banks.^44^

### 2.2 P3HT:PC_61_BM photoactive nanoparticle synthesis

Poly(3-hexylthiophene) (P3HT) (*M*_n_ 20 kDa) and PC_61_BM were synthesised in house (Centre for Organic Electronics, University of Newcastle, Australia) according to literature.^45,46^ Sodium dodecyl sulphate (SDS) with 98% purity was purchased from Sigma-Aldrich. PEDOT:PSS (AI4083) was purchased from Heraeus, Germany and filtered through a 0.45 μm PVDF filter before use. 15 mg of P3HT polymer donor material and 15 mg of PC_61_BM fullerene acceptor material were mixed in 560 μl of anhydrous chloroform and stirred at 500 rpm, 35 °C for 25 min on a hotplate to form organic phase. The aqueous phase was prepared by mixing 33 mg of sodium dodecyl sulphate (SDS) surfactant in 2800 μl of filtered Milli-Q water using stirring condition of 500 rpm for 25 min at room temperature. After that the organic and aqueous phases were mixed to form a macroemulsion and then generate a miniemulsion using Hielscher UP400S (ultra-horn sonication) at 50% amplitude for 2 minutes with a surrounding ice bath in place (to dissipate produced heat during sonication). The miniemulsion was then transferred immediately into the pre-set hotplate (stirrer at 1200 rpm, 60 °C) for overnight to evaporate the chloroform from the emulsion droplets to form the nanoparticle dispersion. Next, the dispersion was placed by pipette into the Hettich Zentrifugen Rotina 420 centrifugal dialysis to remove excess surfactant as well as concentrate the nanoparticle inks following the same procedure reported in our previous work to get an ink solid loading of 6 wt%.^15^

### 2.3 Microscopy

For scanning electron microscopy (SEM) analysis, Na_*x*_WO_3_ nanoparticles were mixed with ZnO in ethanol and SEM samples were prepared by spin coating of 70 μl of the mixture onto glass substrates at 5000 rpm for 1 min. Another sample of mixture of Na_*x*_WO_3_ nanoparticles and ZnO on aluminium sheet was prepared by drop cast of solution to collect the energy dispersive spectrometer (EDS) data. A Zeiss Sigma VP Field Emission SEM (FESEM) was used under 15 kV beam, using back scattering electron detector and magnification ranges of 5k–50k×. High-magnification transmission electron microscopy (TEM) (JEOL JEM-2100 LaB6) instrument also was used to observe the morphology of the ZnO and Na_*x*_WO_3_ NP mixture as well as to collect the local EDS element mapping to validate that the mixture contains both ZnO and Na_*x*_WO_3_ nanoparticles. The acceleration voltage was 200 kV. The TEM sample also prepared by drop cast process on lacey Cu grid and dried the solution in ambient environment. The drop casting of nanoparticles on lacey Cu grid run 2–3 times to confirm enough nanoparticles on grid.

Atomic force microscopy (AFM) images were collected using an Asylum Research Cypher in AC mode. Soft tapping mode Tap150Al-G AFM tips were supplied by Budget Sensors with resonant frequency of 150 kHz and force constant: 5 N m^−1^. Bare ZnO and mixture of Na_*x*_WO_3_ plasmonic nanoparticles with ZnO nanoparticle films were coated onto glass substrates for AFM analysis.

### 2.4 Spectroscopy

To measure the UV-vis spectrum, an ultraviolet-visible absorption spectrophotometer (UV Vis, Varian Cary 6000i) with an integrating sphere was used in the wavelength range of 200–1200 nm in 1 nm step, with a deuterium plasma lamp for the 200–350 nm range, and a tungsten halogen lamp used for the 350–1200 nm range. For photoluminescence (PL) measurements, Shimadzu RF-6000 spectrofluorophotometer with a beam to sample angle of 65°, *λ*_exc_ = 550 nm, *λ*_em_ = 400–900 nm, excitation bandwidth of 5 nm and emission bandwidth of 10 nm was used. A 420 nm high-pass cut-off filter was placed in the path of the beam before the sample. All samples for UV Vis and PL were kept as the same structure of NP-OPV devices (without Al layer).

### 2.5 Device fabrication

To fabricate the NP-OPV devices, pre-cleaned (by water, acetone and isopropanol for 10 minutes) patterned Indium Tin Oxide (ITO) substrates were treated by UV–ozone cleaner for 20 minutes. PEDOT:PSS (AI4083) films of 33 ± 6 nm thickness were spin coated onto ITO at 5000 rpm (1 min) and then dried on a hotplate at 150 °C, 20 min. After that, the PEDOT:PSS coated ITO substrates were treated 10 min into the UV–ozone cleaner. Then, P3HT:PC_61_BM NP ink (35 μl) was spin-coated at 2000 rpm for 1 min and baked for 5 min at 110 °C. The thickness of the photoactive layer was 103 ± 7 nm. Then, ZnO/ZnO : Na_*x*_WO_3_ films were deposited at 5000 rpm for 1 min and dried at 110 °C for 5 min in nitrogen glove box. The thickness of ZnO/ZnO : Na_*x*_WO_3_ layers were measured with an average thickness of 15 ± 6 nm. Finally, Al (thickness: 100 nm) electrodes were deposited under vacuum conditions (10^−6^ torr) *via* thermal evaporation using an Angstrom Amod deposition system.

### 2.6 Device characterisation

The current density–voltage (*J*–*V*) measurements of fabricated NP-OPV devices were conducted using a Newport Class A solar simulator with an AM 1.5 spectrum filter. The light intensity was measured to be 100 mW cm^−2^ by a silicon reference solar cell (FHG-ISE) and the *J*–*V* data were recorded with a Keithley 2400 Source Meter. The NP-OPV devices were masked during testing under AM 1.5 conditions where the masked area was 4 mm^2^. For the device performance, it is reported as the average value ± standard deviation which were calculated from 12 devices based on two substrates with the value for the best devices in the bracket.

## 3 Results and discussion

### 3.1 Characterisation of Na_*x*_WO_3_ and ZnO : Na_*x*_WO_3_ mixed nanoparticles

**Fig. 1:**
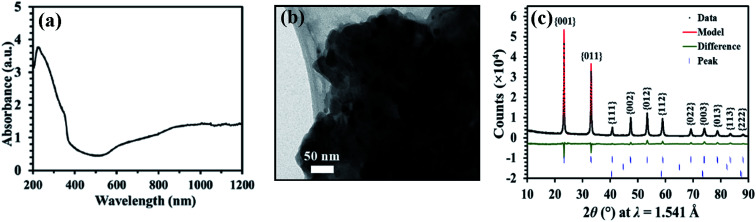
(a) UV-vis spectrum of Na_*x*_WO_3_ nanoparticle film. (b) The TEM image of Na_*x*_WO_3_ nanoparticle with 50 nm scale bar. (c) XRD pattern and Rietveld refinement of Na_*x*_WO_3_ nanoparticles. The black dots show the data, the red line shows the Rietveld model, and the green line is the difference between the two. Peaks of Na_*x*_WO_3_ are indexed according to the *Pm*3̄*m* structure of Straumanis.^41^ The blue dashes indicate the peaks included in the model, from top to bottom corresponding to Na_*x*_WO_3_,^44^ BCC Fe and BCC W.

The light absorption spectra of Na_*x*_WO_3_ nanoparticles coated on a quartz substrate is presented in Fig. 1a. There are two distinct visible and near infrared absorption features arising from the Na_*x*_WO_3_ plasmonic material, at ∼630 nm and ∼1045 nm, corresponding to the excitation of localised surface plasmonic resonances (LSPR) of Na_*x*_WO_3_.^47^ The strong but narrow absorption peak at ∼230 nm in the ultraviolet (UV) range is attributed to the inter-band transition of Na_*x*_WO_3_ plasmonic nanoparticle.^39^ The transmission electron microscopy (TEM) image shown in Fig. 1b indicates that the size of nanoparticles is of the order of tens of nanometres. Fig. 1c shows an XRD pattern of the Na_*x*_WO_3_ nanoparticles after ball-milling. Overall, the refined model is a good fit to the measured data. From quantitative phase analysis, the sample was found to be ≈99 mol% Na_*x*_WO_3_, and ≈1 mol% W and Fe impurity. From the refinement of the Na_*x*_WO_3_ lattice parameter, *a* = 3.8508(1) Å, the Na content was found to be *x* = 0.81.

**Fig. 2:**
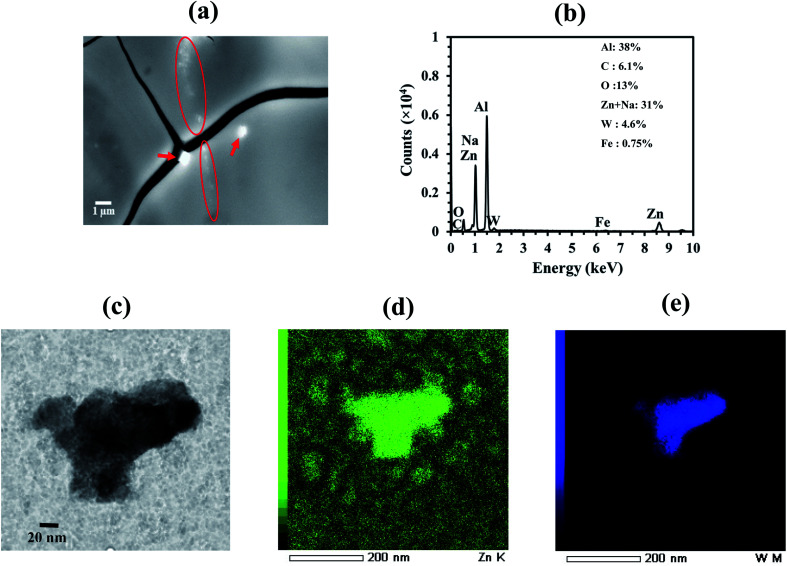
(a) Backscattering detector scanning electron microscopy (BSD-SEM) image of ZnO : Na_*x*_WO_3_ mixture nanoparticles where ZnO : Na_*x*_WO_3_ solution drop casted on Al sheet substrate. The Na_*x*_WO_3_ nanoparticles mixed with ZnO clearly observed in the SEM image those were marked by red arrows and circles. (b) Energy dispersive X-ray spectrum (EDS) of the SEM scanned area indicates the composition of Na, Zn, O and W which confirms the proper mixing of Na_*x*_WO_3_ nanoparticles into the ZnO. (c) High-magnification transmission electron microscopy (TEM) image of the drop cast ZnO : Na_*x*_WO_3_ mixture nanoparticles was presented along with EDS mapping of (d) Zn and (e) W elements.

The back-scattered SEM image of a drop-cast ZnO : Na_*x*_WO_3_ mixture on aluminium sheet is shown in Fig. 2a, with the Na_*x*_WO_3_ nanoparticles clearly visible (marked by arrow sign and circle) as bright objects with respect to background. The EDS spectra (Fig. 2b) clearly show that tungsten is present in the mixture, along with zinc, oxygen and aluminium; consistent with well-mixed ZnO and Na_*x*_WO_3_ plasmonic nanoparticles deposited on an aluminium substrate. The small peaks at about 6.39 keV correspond to the Kα peak of iron; consistent with the low levels of iron impurity identified in the XRD analysis. Moreover, the Zn Lα and Na Kα peaks are overlapped as observed in Fig. 2b, therefore the obtained elemental composition of 31% around 1 keV would be the summation of both elements. However, clear EDS peaks of Zn Kα and W M indicate presence of both Zn and Na in NaWO_3_. High-magnification transmission electron microscopy (TEM) imaging and corresponding Zn and W element EDS mappings of the ZnO : Na_*x*_WO_3_ mixture are shown in Fig. 2c–e. The images show a nanoparticulate aggregate within the ZnO : Na_*x*_WO_3_ mixture, with a Na_*x*_WO_3_ (Fig. 2e) core region surrounded by a ZnO coating (Fig. 2d).

### 3.2 Performance of plasmonic NP-OPV devices

**Fig. 3:**
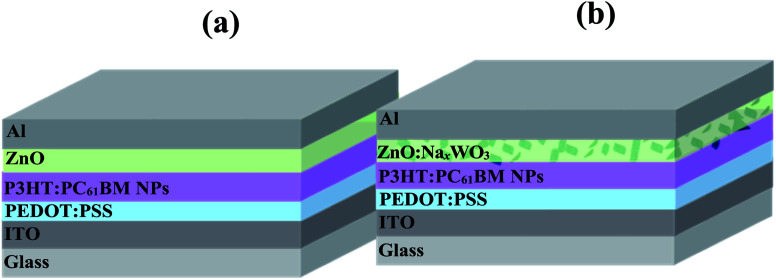
Schematic diagrams of the device architecture (a) control NP-OPV device and (b) Na_*x*_WO_3_ plasmonic NP-OPV device.

The architecture of the control NP-OPV device (ITO/PEDOT:PSS/NP-P3HT:PC_61_BM/ZnO/Al) the plasmonic NP-OPV device (ITO/PEDOT:PSS/NP-P3HT:PC_61_BM/Na_*x*_WO_3_ : ZnO/Al) is shown in Fig. 3a and b, respectively. The Na_*x*_WO_3_ : ZnO weight ratios (dispersed in ethanol) were 0, 0.35, 0.40, 0.45, 0.50.

**Fig. 4:**
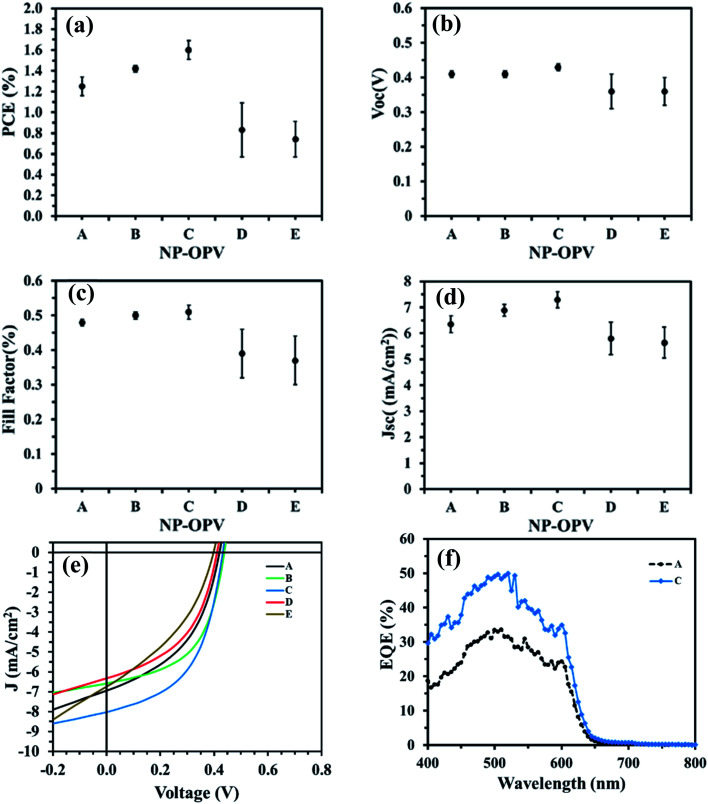
(a) Average power conversion efficiency, (b) average open circuit voltage (*V*_OC_), (c) average fill factor (FF), (d) average short circuit current density (*J*_SC_) and (e) current density–voltage (*J*–*V*) curves of NP-OPV devices with (NP-OPV devices from B to E) and without (NP-OPV device A) Na_*x*_WO_3_ nanoparticles. (f) The external quantum efficiency of the best Na_*x*_WO_3_ NP-OPV device C compared with the control NP-OPV device A.

The performance of the ITO/PEDOT:PSS/NP-P3HT:PC_61_BM/Na_*x*_WO_3_ : ZnO/Al OPV devices (device A to E) for the five different Na_*x*_WO_3_ : ZnO ratios are presented in Fig. 4a–f with the device performance statistics summarised in Table 1. By systematically varying the Na_*x*_WO_3_ : ZnO weight concentration ratios in the suspension based on Table 1 (device A to E), the corresponding device PCEs (Fig. 4a) improved from 1.25 ± 0.09% (1.32%) (device A Na_*x*_WO_3_ : ZnO = 0) to 1.42 ± 0.05% (1.58%) in NP-OPV (device B Na_*x*_WO_3_ : ZnO = 0.35) before maximising at 1.60 ± 0.09% (1.78%) (device C Na_*x*_WO_3_ : ZnO = 0.4). The improvements (corresponding to a ∼17% and ∼35% increase in PCE for device B and C, respectively) originate primarily from an increased FF (Fig. 4c) and *J*_SC_ (Fig. 4d). In particular, for device B, the FF and *J*_SC_ increase from 0.48 to 0.51 and *J*_SC_ from 6.64 mA cm^−2^ to 7.20 mA cm^−2^, respectively, whereas for device C, the FF and *J*_SC_ increase from 0.48 to 0.53 and 6.64 mA cm^−2^ to 7.47 mA cm^−2^ correspondingly. It should be noted that, for device A (control), the 10 mg ml^−1^ of ZnO weight concentration has been optimized to achieve the best OPV device performance.^48^ Therefore, any device performance enhancement cannot be due to the change of ZnO weight concentrations.

**Table 1:** Performance of NP-OPV devices with varied weight concentrations of Na_*x*_WO_3_ nanoparticles mixed in ZnO (from B–E) compare to without (A) Na_*x*_WO_3_ nanoparticles. The average values ± standard deviations that are calculated from 12 devices with the values for the best devices in the bracket

Name of NP-OPV	Weight concentration (mg ml^−1^)		PCE (%)	*V*_OC_ (V)	FF (%)	*J*_SC_ (mA cm^−2^)
	Na_*x*_WO_3_	ZnO				
A	0	10	1.25 ± 0.09	0.41 ± 0.01	0.47 ± 0.02	6.47 ± 0.48
			(1.32)	(0.41)	(0.48)	(6.64)
B	3.5	6.5	1.42 ± 0.05	0.42 ± 0.01	0.50 ± 0.02	6.80 ± 0.16
			(1.58)	(0.43)	(0.51)	(7.20)
C	4	6	1.60 ± 0.09	0.43 ± 0.01	0.51 ± 0.02	7.29 ± 0.31
			(1.78)	(0.45)	(0.53)	(7.47)
D	4.5	5.5	0.83 ± 0.26	0.36 ± 0.05	0.39 ± 0.07	5.80 ± 0.63
			(1.22)	(0.41)	(0.47)	(6.33)
E	5	5	0.74 ± 0.17	0.36 ± 0.04	0.37 ± 0.07	5.64 ± 0.59
			(0.93)	(0.39)	(0.40)	(5.95)

Further increases of the Na_*x*_WO_3_ : ZnO ratio had a negative effect on NP-OPV performance; for device D (Na_*x*_WO_3_ : ZnO = 0.45) and device E (Na_*x*_WO_3_ : ZnO = 0.5) the PCE dropped to 0.83 ± 0.26% (1.22%) and 0.74 ± 0.17% (0.93%), respectively. Devices A to C show no noticeable changes in the trend of *V*_OC_ as presented in Fig. 4b. By contrast, for Na_*x*_WO_3_ : ZnO ratios above 0.4, the PCE starts to decrease as shown in Fig. 4a, due to decreases across *V*_OC_, FF and *J*_SC_ (Fig. 4b–d). The external quantum efficiency (EQE) spectra of best NP-OPV device (device C) in comparison with the control NP-OPV (device A) device are presented in Fig. 4f. Device C exhibits higher EQE compared to device A across the entire spectral range; consistent with the *J*_SC_ data.

### 3.3 Effect of Na_*x*_WO_3_ on device performance

**Fig. 5:**
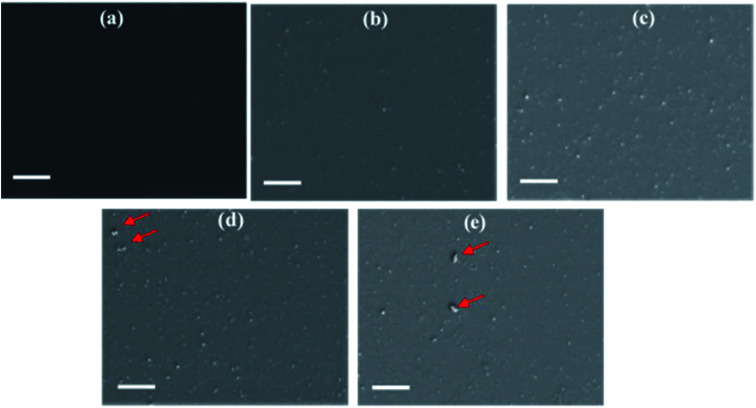
SEM images of mixture of films (prepared on glass substrates) with (a) 0 mg ml^−1^, (b) 3.5 mg ml^−1^, (c) 4 mg ml^−1^, (d) 4.5 mg ml^−1^ and (e) 5 mg ml^−1^ weight concentration of Na_*x*_WO_3_ in ZnO. All of the SEM images are collected using back scattering electron detector. The scale bar is 10 μm.

To investigate the origin of device performance enhancement of Na_*x*_WO_3_ nanoparticle embedded aqueous NP-OPV devices, microscopy and spectroscopy have been used to examine the film quality and the morphological distribution of the plasmonic NP in the overall electron transport layer (ETL). The back scattering SEM images of bare ZnO and Na_*x*_WO_3_ : ZnO films are shown in Fig. 5, with the bright particles (with respect to the background) corresponding to Na_*x*_WO_3_ nanoparticles. Fig. 5a reveal a very smooth bare ZnO film whereas Fig. 5b and c show that for Na_*x*_WO_3_ : ZnO ratios of 0.35 and 0.4, the surface contains an increasing number of Na_*x*_WO_3_ NPs that are evenly distributed within the ZnO film. For Na_*x*_WO_3_ : ZnO ratios of 0.45 (Fig. 5d) and 0.5 (Fig. 5e), Na_*x*_WO_3_ NPs aggregates (marked as red arrow signs) start to appear in the SEM images. The presence of aggregated Na_*x*_WO_3_ nanoparticles in the ZnO ETL would have a detrimental effect on the morphology of the ZnO ETL and, consequently, on the film quality of the subsequently deposited aluminium electrode. As such, the observation of Na_*x*_WO_3_ NP aggregates at Na_*x*_WO_3_ : ZnO ratios above 0.4 is entirely consistent with the decreased device performance observed for devices with higher Na_*x*_WO_3_ loadings.

**Fig. 6:**
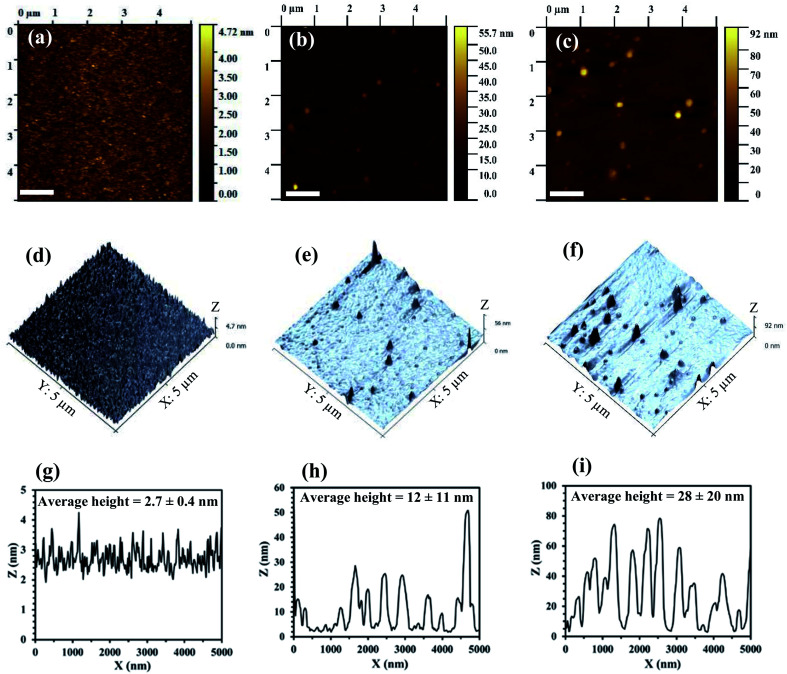
(a)–(c) Show the AFM images (5 μm × 5 μm) of ZnO films with Na_*x*_WO_3_ : ZnO concentration ratios of 0, 0.4 and 0.5, respectively. (d)–(f) Show the corresponding 3D images of (a)–(c). (g)–(i) Typical surface height profiles of the Na_*x*_WO_3_ nanoparticles observed in (a)–(c) with respect to the *X*-axis.

Atomic force microscopy (AFM) has also been used to examine the morphology of a control ZnO film (Fig. 6a), and Na_*x*_WO_3_ : ZnO films with weight ratios of 0.4 (Fig. 6b) and 0.5 (Fig. 6c). In addition, to compare the effect of optimal (Na_*x*_WO_3_ : ZnO = 0.4) and highest Na_*x*_WO_3_ NP concentration (Na_*x*_WO_3_ : ZnO = 0.5) of Na_*x*_WO_3_ on ZnO film, the 3D views of the films are illustrated in Fig. 6d–f. The morphology of bare ZnO film (Fig. 6a) is very smooth, consistent with the SEM image (Fig. 5a) and the surface root-mean-square (RMS) roughness is very small (0.5 ± 0.1 nm). By comparison, the roughness of the Na_*x*_WO_3_ : ZnO = 0.4 film increases to 2.5 ± 2.1 nm. However, the distribution of Na_*x*_WO_3_ nanoparticles in the Na_*x*_WO_3_ : ZnO = 0.4 film (Fig. 6b–e) is uniform, with well separated NPs showing no significant aggregation in the 3D image (Fig. 6e); consistent with the corresponding SEM image (Fig. 5c). By contrast, the surface of Na_*x*_WO_3_ : ZnO = 0.5 film is rougher (RMS 5.5 ± 3.4 nm) with more densely packed Na_*x*_WO_3_ nanoparticles and larger aggregates observed in 3D view (Fig. 6f); consistent with the observation in Fig. 5e. Typical surface height profiles for the three films are presented in Fig. 6g–i, with average NPs heights of 2.7 ± 0.4 nm, 12 ± 11 nm and 28 ± 20 nm for the Na_*x*_WO_3_ : ZnO = 0, 0.4 and 0.5 films, respectively. The AFM data supports the SEM observation that for Na_*x*_WO_3_ : ZnO ratios above 0.4, the Na_*x*_WO_3_ plasmonic nanoparticles start to aggregate and form larger surface structures with dimensions that are large enough to completely penetrate into the aqueous photoactive layer and degrade of device performance. In addition, the increased roughness at ZnO/Al interface can also deteriorate electron transport within NP-OPV device again leading to lower device performance. Therefore, the device performance starts to drop at Na_*x*_WO_3_ : ZnO ratios above 0.4. The AFM morphology of the Na_*x*_WO_3_ : ZnO = 0.35 and Na_*x*_WO_3_ : ZnO = 0.45 samples provide further support for this observation (Fig. S1, ESI†).

**Fig. 7:**
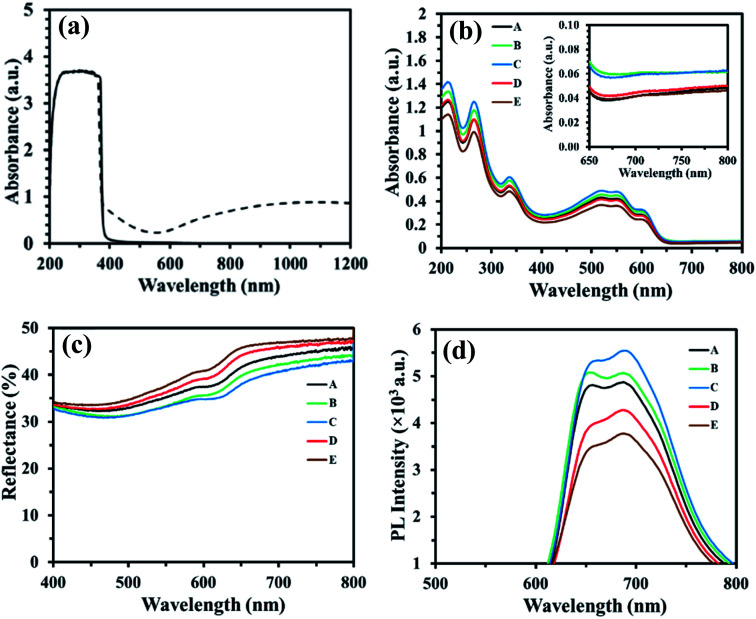
Light absorbance spectra of (a) ZnO : Na_*x*_WO_3_ film (dash line) and ZnO film (solid line). (b) The light absorbance spectrum of PEDOT:PSS/P3HT:PC_61_BM NP/ZnO films A to E, the inset figure expands and highlights the light absorbance spectra in the higher wavelength of visible region. (c) The total light reflectance (specular and diffuse reflectance) spectra and (d) photoluminescence (PL) spectra of the PEDOT:PSS/P3HT:PC_61_BM NP/ZnO films for the control multilayer sample A and PEDOT:PSS/P3HT:PC_61_BM NP/ZnO : Na_*x*_WO_3_ samples B to E.

The UV-vis absorption spectra of the ZnO and the optimal Na_*x*_WO_3_ : ZnO ratio (0.4) films on quartz substrates are presented in Fig. 7a. It clearly shows the light absorption arising from the Na_*x*_WO_3_ NPs across the visible to near infrared regions compared to the ZnO only film and matches the light absorption spectra of the pure Na_*x*_WO_3_ film shown in Fig. 1a. The UV-vis data confirms that the Na_*x*_WO_3_ : ZnO film consists of well-mixed Na_*x*_WO_3_ nanoparticles within the ZnO layer; consistent with the EDS data in Fig. 2. The absorption spectra of the PEDOT:PSS/NP-P3HT:PC_61_BM/ZnO multilayer films with and without Na_*x*_WO_3_ nanoparticles are presented in Fig. 7b, where the light absorption in the near infrared range (650–800 nm) is presented as an inset. Sample A to E follow the same Na_*x*_WO_3_ : ZnO ratios described in Table 1. The effect of the Na_*x*_WO_3_ plasmonic enhancement on the light absorption is most clearly visible for samples B and C. As the weight concentration of Na_*x*_WO_3_ NPs in the ZnO film is increased, the light absorbance of sample B and C across the visible region is enhanced by around 9% and 15%, respectively, compared to the control sample A. This increased light absorption explains the enhanced photocurrent in the plasmonic nanoparticle embedded NP-OPV devices of B and C over the control NP-OPV device. The higher scattering background in the near infrared range of 650 to 800 nm (inset) for samples B and C is also consistent with a plasmonic enhancement due to light scattering. Furthermore, the overall light absorption of the plasmonic nanoparticle embedded films for sample D and E decreases by around 1% (sample D) and 11% (sample E) as the Na_*x*_WO_3_ : ZnO ratio increases to 0.45 and 0.5, respectively, again in good agreement with the variation of the photocurrent density.

To further probe the effect of the higher Na_*x*_WO_3_ weight concentrations on light absorption, the total (specular plus diffuse) light reflectance spectrum of PEDOT:PSS/NP-P3HT:PC_61_BM/ZnO : Na_*x*_WO_3_ multilayer films were collected and are presented in Fig. 7c. Samples D and E exhibit higher reflectivity across the entire 400–800 nm spectral range, consistent with decreased light absorption^32,49^ arising from the increased aggregation of the Na_*x*_WO_3_ NPs observed for these samples; resulting in a reduced plasmonic enhancement of their surrounding electric field.^50^ Moreover, as the aggregated NPs are large enough (peak heights > 50 nm observed in Fig. 6i) to penetrate the active layer, it is likely that the morphology of the water processed P3HT:PC_61_BM nanoparticle based photoactive layer is also degraded; decreasing the light absorption capacity of these multilayer films D and E. By contrast, the spectrum-wide lower reflectivity of the lower Na_*x*_WO_3_ : ZnO ratio samples (B and C) is consistent with increased light absorption arising from the plasmonic enhancement of the Na_*x*_WO_3_ NPs.

The photoluminescence (PL) spectra of the PEDOT:PSS/NP-P3HT:PC_61_BM/ZnO (Na_*x*_WO_3_) multilayer films are presented in Fig. 7d in order to investigate the effect of Na_*x*_WO_3_ nanoparticles on exciton generation. The incorporation of the Na_*x*_WO_3_ plasmonic NPs leads to broader PL spectra and an increase of the PL intensity by ∼8% for the multilayer film B and by ∼13% for the multilayer film C compared to the control multilayer film A. The broadening and enhancement of the PL intensity can be attributed to the fact that the LSPR excitation and light scattering of the Na_*x*_WO_3_ plasmonic nanoparticles inside the device increase the degree of light collection, thereby, leading to an enhanced light excitation generation rate.^51^ Moreover, the enhanced PL intensity arises from the strong coupling between the excitonic state of the polymer and the plasmonic field of the Na_*x*_WO_3_ nanoparticles, which is due to the oscillation of the surface plasmonic dipole electric field and excitons.^52^ Thus, it can be concluded that the incorporation of Na_*x*_WO_3_ nanoparticles significantly enhances the exciton generation, rate as observed in multilayer films B and C. However, further increasing the Na_*x*_WO_3_ nanoparticles weight concentration in the ZnO results in the aggregation of nanoparticles, as is evident in the SEM images of multilayer films D and E (Fig. 5d and e). As a result, the electric field intensity starts to decay with the increase of nanoparticle aggregate size, leading to a reduction in the LSPR excitation effect of the plasmonic materials, as well a decrease in the PL intensity of the multilayer film, as shown in Fig. 7d, which is consistent with the UV-vis spectra of the corresponding multilayer films D and E.

**Fig. 8:**
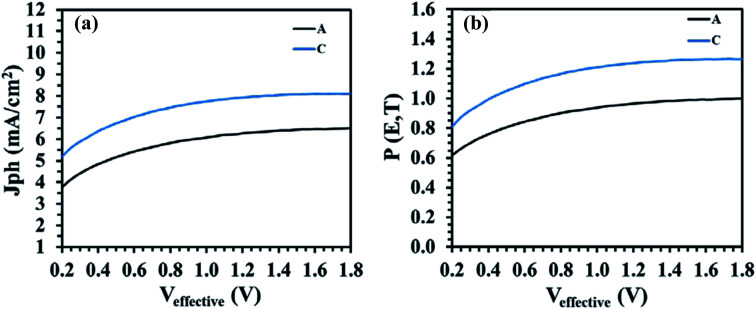
(a) *J*_ph_*vs. V*_eff_ curves and (b) *P*[*E*,*T*] *vs. V*_eff_ curves of the NP-OPV devices with (NP-OPV device C) and without (NP-OPV device A) Na_*x*_WO_3_ plasmonic nanoparticles.

To further validate the effect of Na_*x*_WO_3_ nanoparticles on the exciton generation and dissociation of the NP-OPV devices, the maximum exciton generation rate (*G*_max_) and the exciton dissociation probability (*P*[*E*,*T*]) are considered for the hero NP-OPV device C with respect to control NP-OPV device A, following a previously reported method.^32,53,54^ The dependence of the photocurrent density (*J*_ph_) on the effective voltage (*V*_eff_) is shown in Fig. 8a for NP-OPV device A and NP-OPV device C. Herein, *J*_ph_ is calculated by the following formula:

1 *J*_ph_ = *J*_L_ − *J*_D_

where, *J*_L_ is the current densities under light and *J*_D_ is the current densities under dark.

*V*_eff_ is described by the following equation:

2 *V*_eff_ = *V*_0_ − *V*_a_

where, *V*_0_ is the voltage when photocurrent density (*J*_ph_) equals to zero and *V*_a_ is the applied voltage. As illustrated in Fig. 8a, *J*_ph_ increases with increasing of *V*_eff_ and then approaches a saturation limit at high effective voltages. The value of the saturation photocurrent density *J*_sat_,^35^ is independent of the bias and temperature and can be derived from Fig. 8a directly. Assuming that all the photo-generated excitons are dissociated and contribute to the current at high *V*_eff_, *G*_max_ can also be calculated from:

3 *J*_sat_ = *qG*_max_*L*

where, *q* is the electronic charge and *L* is the thickness of the active layer (here, *L* = 103 nm). The resulting *G*_max_ values for the control NP-OPV device A and the plasmonic NP-OPV device C were 5.12 × 10^28^ m^−3^ s^−1^ (*J*_sat_ = 8.10 mA cm^−2^) and 4.15 × 10^28^ m^−3^ s^−1^ (*J*_sat_ = 6.52 mA cm^−2^), respectively. The impressive enhancement (∼21.5%) in *G*_max_ strongly suggests that the presence of the Na_*x*_WO_3_ nanoparticles in NP-OPV device C amplifies the exciton generation rate in the photoactive layer. As the maximum exciton generation rate (*G*_max_) is related to the maximum absorption of incident light,^53,55^ the enhancement *G*_max_ implies increased light absorption in NP-OPV device C due to the plasmonic nanoparticles, which in good agreement with the observed increased light absorption of around 15% in the corresponding absorbance spectra (Fig. 7b). In addition, to measure the effective charge separation, the exciton dissociation probability, *P*[*E*,*T*] was also calculated and is presented with respect to the effective voltage (*V*_eff_) in Fig. 8b. To calculate the exciton dissociation probability *P*[*E*,*T*] under the short-circuit condition (*V*_a_ = 0) the following equation is used:

4 *J*_ph_ = *qG*_max_*P*[*E*,*T*]

The corresponding values of *P*[*E*,*T*] at any effective voltage (*V*_eff_) can be attained from the plot of the normalized photocurrent density *J*_ph_/*J*_sat_. The calculated *J*_ph_ values at *V*_a_ = 0 were 4.88 mA cm^−2^ and 6.40 mA cm^−2^ resulting in *P*(*E*,*T*) of around 75% and 80%, for the control (NP-OPV device A) and best Na_*x*_WO_3_ (NP-OPV device C) devices, respectively. These results demonstrate that the presence of the Na_*x*_WO_3_ nanoparticles in the NP-OPV structure directly increases the dissociation of excitons into free carriers.

For the best of our knowledge, our work is the first study using plasmonic nanoparticles, in particular, a plasmonic material other than Au or Ag nanostructures, in aqueous processed OPV devices. We put a summary of literature in Table 2 that shows different types of plasmonic nanostructures used in bulk heterojunction (BHJ) OPV devices and compare them with our aqueous processed OPV work. The enhancement due to the plasmonic nanostructure observed in this work is comparable to those studies. It also should be noted that the previous studies in our group have showed that the aqueous processed OPV devices behave very differently from the conventional BHJ OPV devices.^12–15^ This work points a future direction to further explore Na_*x*_WO_3_ nanoparticles in the OPV applications.

**Table 2:** A summary of reported organic photovoltaic devices with different plasmonic materials or nanostructures. The bracket values refer to the control device without plasmonic nanostructure

Plasmonic structure	Location of plasmonic structure	Proposed mechanism	PCE (%)	*J*_SC_ (mA cm^−2^)	Enhancement (%)	
					PCE	*J*_SC_
Au nano-rods^32^	ETL	Scattering	8.01 (7.43)	17.17 (16.27)	7.8	5.5
Fe_3_O_4_ NPs^33^	Photoactive layer	Scattering	2.22 (1.09)	8.62 (7.74)	104	11
Au–Ag nano-cube^35^	HTL	Electric field and scattering	3.10 (2.85)	8.70 (8.40)	8.8	3.6
Au NPs^36^	HTL	LSPR	4.19 (3.48)	10.18 (8.95)	20	14
Au spheres and Au rods^37^	HTL	Scattering and LSPR	4.28 (3.46)	11.49 (9.28)	24	24
Au spheres and GO composites^38^	HTL	Near field effect	3.98 (3.25)	10.44 (9.37)	22	11
Au nano-arrows^56^	ETL	Scattering and LSPR	7.82 (6.14)	17.40 (14.70)	27	18
Au NPs–TiO_2_ composite^57^	ETL	Scattering and LSPR	4.20 (3.73)	10.15 (9.38)	13	8.2
Au/Ag NPs^58^	Active layer	LSPR	4.85 (4.44)	12.21 (11.17)	9.2	9.3
Ag NPs^59^	Active layer	LSPR	3.92 (3.19)	10.41 (8.67)	23	20
Ag NPs–SiO_2_ ^60^	Active layer	LSPR	3.96 (3.44)	9.50 (8.37)	15	14
Au NPs–SiO_2_ ^61^	Active layer	LSPR	2.17 (1.95)	9.19 (7.23)	11	27
Au NRs^62^	Active layer	LSPR	3.58 (3.17)	8.73 (7.97)	13	9.5
Na_*x*_WO_3_ [this work]	ETL	Scattering and LSPR	1.78 (1.32)	7.47 (6.64)	35	13

## 4 Conclusions

Low cost Na_*x*_WO_3_ plasmonic nanoparticles have been added to the architecture of water processed NP-OPVs and an improved device performance is found. By carefully controlling the mixing ratio of the absolute weight concentration of Na_*x*_WO_3_ (4 mg ml^−1^) and ZnO in the cathodic interfacial layer, the light absorbance of the NP-OPV device can be improved about 15% which leads to an enhanced EQE. The *J*_SC_ of the best NP-OPV device is found to be amplified about 12.5% and ultimately, the PCE of the corresponding NP-OPV device was boosted from 1.32% (1.25 ± 0.09%) (control NP-OPV device without Na_*x*_WO_3_) to 1.8% (1.60 ± 0.09%) (with Na_*x*_WO_3_), about a 35% enhancement. Further investigation of the maximum exciton generation rate and probability of exciton dissociation of this hero NP-OPV device showed that these parameters increased by around 21.5% and 6.6% correspondingly, confirming the effect of Na_*x*_WO_3_ nanoparticles on enhancing the performance of NP-OPV device. In addition, recorded PL data is consistent with the calculated maximum exciton generation rate at the optimized mixing ratio of ZnO : Na_*x*_WO_3_ used in the NP-OPV devices. The overall results indicate that enhancement in light absorption of the water processed photoactive layer by scattering and LSPR effects increases the exciton generation rate and probability of exciton dissociation which play the most important roles in the charge generation of the water processed NP-OPV device. However, our current study so far can rule out the possibility that the device performance improvement is due to morphological difference(s) in the photoactive layer, and/or, the interfacial layer between the photoactive layer and the Na_*x*_WO_3_ embedded ZnO buffer layer. Since this is the first study on Na_*x*_WO_3_ based aqueous processed OPVs, further work to clarify the plasmonic effect is warranted.

## Conflicts of interest

There are no conflicts to declare.

## Supplementary Material

RA-011-D1RA02328D-s001
