# Supplementary material for: Plasmonic enhancement of aqueous processed organic photovoltaics
Source: RSC Adv. 2021 May 25;11(31):19000–11. doi: 10.1039/d1ra02328d (PMC9033506; doi:10.1039/d1ra02328d)
Supplement: RA-011-D1RA02328D-s001 [file RA-011-D1RA02328D-s001.pdf]

Electronic Supplementary Material (ESI) for RSC Advances.
This journal is © The Royal Society of Chemistry 2021

Supporting Documents

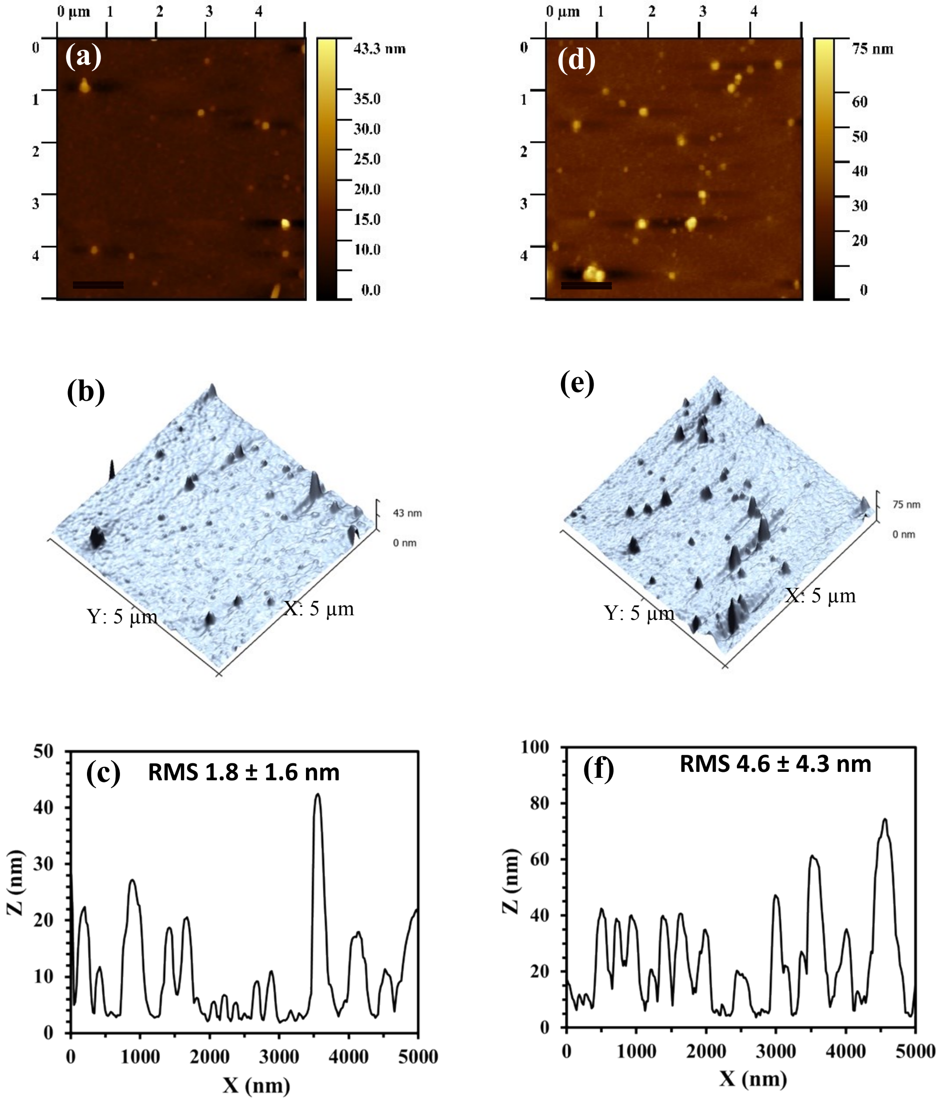

Figure S1: The AFM morphology images (5µm x 5µm) of ZnO films with (a) 3.5 mg/ml and (d) 4.5 mg/ml weight concentrations of plasmonic Na_xWO_3 nanoparticles. The 3D images of (b) 3.5 mg/ml and (e) 4.5 mg/ml weight concentrations of Na_xWO_3 nanoparticles embedded ZnO films showing the surface morphology as well as height of each plasmonic nanoparticle. The calculated height of each plasmonic nanoparticle along with X-axis are presented for (c) 3.5 mg/ml and (d) 4.5 mg/ml weight concentrations of plasmonic Na_xWO_3 nanoparticles based ZnO films where the average height of all nanoparticles for corresponding films are 10 ± 8.5 nm and 24 ± 17 nm.
